# Effect of Injection Molding on the Performance of EDTAD-Functionalized Oat Protein Bioplastics as Water-Absorbent Materials

**DOI:** 10.3390/polym18192338

**Published:** 2026-09-25

**Authors:** María Vázquez-Ortego, Patricia Morales, Barbara Tomadoni, Carlos Bengoechea

**Affiliations:** Departamento de Ingeniería Química, Escuela Politécnica Superior, Universidad de Sevilla, C/Virgen de África, 7, 41011 Sevilla, Spain; mvazquez14@us.es (M.V.-O.); cbengoechea@us.es (C.B.)

**Keywords:** oat protein, protein functionalization, injection molding, water-absorbent bioplastics, water uptake capacity

## Abstract

Sustainable water-absorbent materials derived from renewable resources represent an attractive alternative to petroleum-based counterparts. Oat protein concentrate (OPC), an underutilized plant protein, has considerable potential for this purpose, although its processing into injection-molded bioplastics has not previously been reported. While chemical functionalization has been explored to enhance the water affinity of protein-based materials, the combined influence of functionalization and thermomechanical processing on oat protein bioplastics remains unexplored. In this study, OPC was functionalized with ethylenediaminetetraacetic dianhydride (EDTAD) and processed by injection molding to investigate the combined effects of formulation, chemical functionalization, and mold temperature on the rheological, mechanical, thermal, and water-absorption properties of the resulting bioplastics. Among the formulations evaluated with glycerol (Gly) as plasticizer, OPC/Gly-70/30 showed the best balance between mechanical performance, water uptake, and soluble matter loss and was selected for functionalization. FTIR analysis showed spectral changes consistent with the introduction of additional carboxyl-containing groups after EDTAD treatment. Functionalization reduced the viscoelasticity of the protein/Gly doughs before processing but increased the viscoelastic moduli of the injection-molded bioplastics, showing that the effect of functionalization on the viscoelastic response depended on the processing stage. Although functionalized bioplastics molded at 120 °C exhibited lower water uptake than the native materials, reducing the mold temperature to 60 °C increased their water uptake capacity from approximately 115% to 232%, whereas the native bioplastics remained essentially unchanged. These findings highlight the interplay between chemical functionalization and thermomechanical processing, with mold temperature emerging as a key design parameter. The enhanced water uptake was accompanied by a reduction in tensile properties, highlighting the need to balance absorbency and mechanical performance when developing these materials for specific applications.

## 1. Introduction

Water-absorbent materials are widely used in personal care, agriculture, food packaging, and biomedical applications owing to their ability to retain large amounts of water while maintaining structural integrity [1]. Most commercial absorbent materials are based on petroleum-derived polymers, raising concerns regarding biodegradability, fossil resource consumption, and microplastic generation. Several synthetic polymers are currently used to produce absorbent materials, particularly polyacrylamide, poly(acrylic acid), and their derivatives [2]. These synthetic materials are not biodegradable and can release harmful chemicals when they are in contact with water or degraded, thereby causing harm to wildlife and ecosystems [3]. Hence, there is growing interest in developing sustainable absorbent materials from renewable biopolymers with tailored structure and functionality.

Among the different biopolymers researched, proteins constitute an attractive alternative because of their biodegradability and the possibility of tailoring their functionality through chemical modification [4,5]. Oat protein (OP) is particularly interesting because it is an underexploited by-product generated during oat fractionation for starch, β-glucan, and oat-based beverages [6]. Global oat production has remained relatively stable in recent years, at approximately 23 million tons per year, being mostly used for animal feed [7]. Therefore, its valorization represents an opportunity to develop sustainable, value-added materials while reducing agro-industrial waste. Although injection molding has been successfully employed to manufacture bioplastics from several protein sources, including soy, pea, and rice proteins, its application to oat protein-based bioplastics remains unexplored. To the best of our knowledge, no studies have reported the production of oat protein-based bioplastics through injection molding, despite the industrial relevance and scalability of this processing technology.

To improve the water uptake of oat protein, chemical functionalization through acylation represents an effective strategy to increase its water-binding capacity. Previous studies have demonstrated that introducing carboxylic acid groups into soy proteins significantly enhances their water absorption capacity, suggesting that a similar approach could be successfully applied to oat proteins [8]. During acylation, the amino groups of the protein, mainly those of lysine residues, react with the acylating agent to form amide bonds while introducing additional carboxyl groups, thereby increasing the hydrophilicity of the protein [9]. Since oat protein has a relatively high lysine content (3.7%) [10], it is considered a promising candidate for this type of modification. In this study, ethylenediaminetetraacetic dianhydride (EDTAD) was selected as the acylating agent, which generates low-toxicity by-products during the functionalization process, resulting in a reduced environmental impact compared with conventional synthetic alternatives [9]. Among the available acylating agents, EDTAD is particularly attractive because each reaction introduces three additional carboxyl groups through the opening of the dianhydride ring, as illustrated in Figure 1, leading to a greater potential increase in water uptake [9].

Although chemical functionalization has proven effective for increasing the water affinity of several protein-based materials, the final performance of these materials also depends on the processing method used to consolidate the polymer network. Thermomechanical processes, such as injection molding, promote protein denaturation, chain rearrangement, and ultimately, new intermolecular interactions, which may enhance or counteract the intended effects of the chemical modification. While injection molding has been investigated for other plant protein systems, its application to oat protein remains unexplored, and the influence of processing conditions on EDTAD-functionalized oat protein materials has not been reported. Consequently, the combined effects of protein functionalization and thermomechanical processing on oat protein-based bioplastics remain unknown. In particular, it remains unclear whether processing enhances or suppresses the benefits of chemical functionalization. To address this question, the present work investigates how injection molding influences the performance of EDTAD-functionalized oat protein bioplastics. Specifically, the effects of formulation, chemical functionalization, and mold temperature on the rheological, thermal, mechanical, and water uptake properties were systematically evaluated to elucidate the interplay between chemical modification and thermomechanical processing in the design of sustainable water-absorbent materials.

## 2. Materials and Methods

### 2.1. Materials

The oat protein concentrate (OPC) used in this study was kindly provided by Lantmännen AB (Stockholm, Sweden). The protein content was determined from the total nitrogen content using a LECO CHNS-932 Nitrogen Analyzer (Leco Corporation, St. Joseph, MI, USA) and a nitrogen-to-protein conversion factor of 6.25, resulting in a protein content of 55.3 wt%. The isoelectric point (pI) was determined using the Markwell method [11,12] and was found to be 3.5. Ethylenediaminetetraacetic dianhydride (EDTAD), used as an acylation agent, was purchased from Sigma-Aldrich (St. Louis, MO, USA). Glycerol (Gly), used as a plasticizer, was supplied by PANREAC Química S.A. (Barcelona, Spain).

### 2.2. Functionalization with EDTAD

To functionalize oat protein, acylation with EDTAD was performed following the method of Cuadri et al. [8], with modifications. Briefly, a 4.5% (*w*/*v*) OPC solution was prepared in distilled water, and the pH was adjusted to 12 using 6 M NaOH. EDTAD was then added to obtain an EDTAD/OPC ratio of 0.12. The pH was maintained at 12 for 1 h by adding 1 M NaOH as needed. After 1 h, the reaction mixture was neutralized to pH 7 using 5 M HCl. Then, the reaction mixture was transferred into dialysis bags and immersed in distilled water at a solution-to-water ratio of 1:20 to initiate the dialysis process [8]. The dialysis water was renewed every 24 h.

### 2.3. Fourier Transform Infrared Spectroscopy (FTIR)

IR spectra were recorded in the 4000–400 cm^−1^ wavenumber range with 2 cm^−1^ resolution and 32 scans using a Bruker-Invenio-X FTIR spectrometer (Billerica, MA, USA) equipped with an attenuated total reflectance (ATR) accessory. Samples were analyzed directly with the ATR instrument, without KBr pellet preparation, in the transmittance mode. The data were then normalized and converted to absorbances for each sample. The resulting spectra are reported in normalized absorbance units versus wavenumbers.

### 2.4. Processing

#### 2.4.1. Mixing Stage

Homogeneous doughs (protein concentrate + plasticizer) were prepared by mixing OPC and Gly in different ratios (50/50, 60/40, and 70/30) using a Haake Rheomix QC batch mixer for the Haake Polylab QC system (ThermoHaake, Karlsruhe, Germany) for 10 min at a speed of 50 rpm. Temperature and torque values were recorded throughout the mixing process inside the mixer cavity [13].

#### 2.4.2. Injection Molding

The resulting OPC/Gly doughs were processed by injection molding using a MiniJet II Piston Injection Molding System (Thermo Scientific, Erlangen, Germany). The mold temperature (120 °C), cylinder temperature (40 °C), injection pressure (500 bar), post-injection pressure (500 bar), and residence time in the chamber (180 s) were selected based on preliminary experiments and kept constant throughout the study. For water uptake experiments, mold temperature was evaluated as a processing variable (varying from 60 to 140 °C). Rectangular specimens (60 mm × 10 mm × 1 mm) were prepared.

### 2.5. Characterization of Materials

#### 2.5.1. Dynamic Mechanical Analysis (DMA)

Dynamic mechanical analysis (DMA) was performed to characterize the viscoelastic properties of the OPC/Gly doughs and the resulting bioplastics. Measurements were carried out in duplicate using a Discovery DMA 850 dynamic mechanical analyzer (TA Instruments, New Castle, DE, USA). For each formulation, the reported replicates corresponded to specimens obtained from the same processing batch.

The OPC/Gly doughs were analyzed using a compression clamp, whereas the molded bioplastics were tested using a film tension clamp. All measurements were conducted within the linear viscoelastic region (LVR), previously determined by strain sweep tests.

Frequency-sweep tests were performed at 20 °C over a frequency range of 0.1–100 Hz to determine the storage modulus (E′) and loss modulus (E″) as a function of frequency. Temperature ramp tests were carried out from 0 °C to 150 °C for doughs and −30 °C to 150 °C for bioplastics, at a heating rate of 3 °C min^−1^ and a constant frequency of 1 Hz to evaluate the variation of E′ and E″ with temperature.

#### 2.5.2. Tensile Test

To estimate the mechanical properties of the bioplastics, including tensile strength, elongation at break, and Young’s modulus, tensile tests were performed on rectangular specimens at room temperature using the RSA III Rheometrics System Analyzer (TA Instruments, New Castle, DE, USA) operating in tension mode. Specimens were conditioned in a chamber at constant humidity and temperature (50% relative humidity, room temperature) for 3 days. The specimens were stretched at a crosshead speed of 1 mm·min^−1^ until failure [14]. At least six specimens obtained from the same processing batch were evaluated for each formulation.

#### 2.5.3. Water Uptake Capacity (WUC) and Soluble Matter Loss (SML)

Water uptake capacity (WUC) was determined according to the UNE-EN ISO 62:2008 standard [15]. Rectangular specimens obtained from the same processing batch were cut into three equal sections and dried in an oven at 50 °C for 24 h. The initial dry weight was recorded as $w_{0}$. The dried specimens were then immersed in distilled water for 24 h, removed, gently blotted to eliminate surface water, and weighed ($w_{1}$). Subsequently, the samples were dried again at 50 °C for 24 and weighed to obtain the final dry weight ($w_{2}$). WUC was calculated using Equation (1).

The loss of soluble material was determined using Equation (2) by comparing the initial dry weight ($w_{0}$) with the final dry weight after immersion and redrying ($w_{2}$).

(1) $$WUC\;\left(\%\right)=\frac{w_{1}-w_{2}}{w_{2}}\cdot 100$$

(2) $$SML\;\left(\%\right)=\frac{w_{0}-w_{2}}{w_{0}}\cdot 100$$

### 2.6. Statistical Analysis

The data were analyzed using the open-source R Statistical Software v. 4.3.2 [16]. For the comparison of the different OPC/Gly formulations, analysis of variance (ANOVA) followed by Tukey–Kramer multiple comparisons was performed (*p* < 0.05). For the mold-temperature experiment, two-way ANOVAs were performed to assess the effects of functionalization and mold temperature on WUC and SML, including their interaction. When significant effects were detected, pairwise comparisons were performed using estimated marginal means. The Šidák adjustment was used to compare functionalization treatments at each molding temperature and among molding temperatures within each functionalization treatment. Differences were considered statistically significant at *p* < 0.05.

## 3. Results and Discussion

### 3.1. OPC/Gly System Characterization

#### 3.1.1. DMA

Figure 2 shows the dynamic mechanical properties of the different OPC/Gly systems: OPC/Gly doughs and injection-molded bioplastics.

For the dough systems, frequency-sweep tests revealed that both the storage modulus (E′) and loss modulus (E″) increased with increasing OPC content. However, this increment was more pronounced between the 50/50 and 60/40 formulations than between the 60/40 and 70/30 formulations, the latter exhibiting very similar modulus values. This performance can be attributed to the plasticizing effect of glycerol, which increases the free volume and mobility of protein chains while reducing protein–protein interactions. Consequently, decreasing the glycerol/protein ratio may promote the formation of a more interconnected protein network, resulting in stiffer materials with higher viscoelastic moduli, as previously reported for protein-based systems [17]. The smaller increase observed between the 60/40 and 70/30 formulations suggests that the reinforcing effect of increasing OPC content becomes less pronounced at higher protein concentrations, indicating that the network approaches a saturation state [18]. In all formulations, E′ remained higher than E″ throughout the studied frequency range, indicating a solid-like behavior.

The temperature ramp tests showed the same trend observed in the frequency-sweep tests. E′ remained higher than E″ throughout the entire temperature range, confirming the solid-like behavior of all doughs. Increasing the OPC content also resulted in higher viscoelastic moduli, although the differences between the 60/40 and 70/30 formulations remained much smaller than those between the 50/50 and 60/40 formulations. Despite these similarities, the thermal evolution differed depending on formulation. As temperature increased, both E′ and E″ progressively decreased in the 60/40 and 70/30 doughs, whereas the 50/50 formulation showed only minor changes throughout the studied temperature range. This response is attributed to the higher glycerol content of the 50/50 formulation, suggesting that the higher glycerol content plasticizes the network already at room temperature and reduces the sensitivity of its viscoelastic properties to increasing temperature [19].

For all formulations, tan δ remained below 1 throughout the studied temperature range, confirming the predominance of the elastic response over the viscous one. Although the formulations with higher OPC contents exhibited slightly higher tan δ values, the differences were considerably smaller than those observed for E′ and E″, suggesting that increasing the protein content mainly reinforced the stiffness of the network without substantially altering the balance between its elastic and viscous components. The slightly higher tan δ values observed for the 60/40 and 70/30 formulations may be associated with the greater structural heterogeneity of the protein network, where the coexistence of protein-rich and glycerol-rich domains gives rise to a broader distribution of molecular relaxation processes [20]. A broad tan δ peak was observed for all formulations between approximately 65 and 80 °C, which is commonly associated with the glass transition region in dynamic mechanical analysis [21]. The transition occurred at approximately 65 °C for the 60/40 and 70/30 formulations and at approximately 80 °C for the 50/50 formulation. Although higher plasticizer contents are generally expected to decrease the glass transition temperature, protein–glycerol systems often exhibit multiple relaxation processes associated with the coexistence of protein-rich and glycerol-rich domains. Therefore, the transition observed in the present work most likely corresponds to the relaxation of the protein-rich domains rather than to the glycerol-rich phase, as previously reported by Chen et al. [20].

Oat protein-based bioplastics were successfully obtained through injection molding (Figure 3). The frequency-sweep results (Figure 2C) for the bioplastics followed the same overall trend observed for the corresponding doughs. In all formulations, E′ remained higher than E″, confirming the predominance of the elastic response, while both moduli increased with increasing OPC content, consistent with the formation of a stiffer protein network expected at lower glycerol/protein ratios [17]. However, the differences among formulations were less pronounced than those observed for the doughs, suggesting that the injection molding process partially reduced the influence of the initial composition. Furthermore, all bioplastics presented substantially higher viscoelastic moduli than the corresponding doughs, suggesting that the combined action of temperature, pressure, and shear during injection molding may have promoted structural rearrangements within the protein matrix. Similar improvements in the viscoelastic and mechanical performance of thermally processed protein-based materials have been reported for soy protein systems [22].

The temperature ramp tests (Figure 2D) also revealed a common behavior for all bioplastics. Both E′ and E″ gradually decreased up to approximately 100 °C, after which a slight increase in both moduli was observed. The initial decrease is consistent with the progressive softening of the protein–glycerol matrix as temperature increases, whereas the subsequent increase may be attributed to further rearrangement of the protein network and the formation of additional intermolecular interactions promoted by heating, resulting in a slight reinforcement of the material [23]. As in the frequency-sweep tests, formulations with higher OPC contents showed higher viscoelastic moduli, although the differences among formulations remained smaller than those observed for the doughs, further supporting the homogenizing effect of the injection molding process.

#### 3.1.2. Tensile Test

To determine whether the rheological differences observed after processing translated into structural performance, the mechanical properties of the resulting bioplastics prepared from native OPC were evaluated by tensile testing. The average values of Young’s modulus (E), tensile strength (σ_max_), and elongation at break (ε_max_) are presented in Table 1. The corresponding tensile stress–strain curves are provided in the Supplementary Material (Figure S1).

Young’s modulus and tensile strength showed no significant differences between the 50/50 and 60/40 formulations, whereas both parameters increased significantly for the 70/30 formulation. These results indicate that the reinforcing effect of increasing OPC content became evident only at the highest protein concentration evaluated, where the lower glycerol/protein ratio might promote the formation of a stiffer and stronger protein network through enhanced protein–protein interactions [17,18].

In contrast, elongation at break displayed non-monotonic behavior, reaching its maximum value for the 60/40 formulation and decreasing for both the 50/50 and 70/30 systems. This result suggests that extensibility depends on achieving an adequate balance between protein network integrity and chain mobility. At high protein contents (70/30), the reduced amount of glycerol restricts molecular mobility, producing a rigid network with limited deformability and earlier fracture despite its higher stiffness and strength [24]. Conversely, the higher glycerol content of the 50/50 formulation increases chain mobility but may also reduce the effectiveness of stress transfer within the protein network, leading to lower extensibility than that observed for the 60/40 formulation [25]. Therefore, the intermediate protein/glycerol ratio provided the best compromise between stiffness and flexibility, resulting in the highest elongation at break.

#### 3.1.3. Water Uptake Capacity and Soluble Matter Loss

Regarding the functional properties of the bioplastics, the water uptake capacity (WUC) ranged from 161 to 175%, with no clear compositional trend and a slightly higher value for the 60/40 formulation, as shown in Table 2. This response likely results from the balance between two opposing effects. On one hand, glycerol is highly hydrophilic and promotes water sorption into the matrix. On the other hand, increasing the protein content leads to a more interconnected network that restricts chain mobility and limits matrix swelling. As a result, the enhanced water affinity provided by glycerol is partially compensated by the greater structural constraint imposed by the protein network, resulting in similar WUC values for all formulations [26].

In contrast, soluble matter loss (SML) decreased progressively with decreasing glycerol content (Table 2). Since glycerol is a low-molecular-weight, water-soluble plasticizer that is not covalently bound to the protein matrix, it readily diffuses into the aqueous medium during immersion. Consequently, formulations containing higher amounts of glycerol exhibited greater SML values. Moreover, the more interconnected protein network developed at higher OPC contents may further hinder the diffusion of soluble compounds, contributing to the lower SML observed for the 70/30 formulation. This behavior agrees with previous studies reporting that SML in protein-based bioplastics is primarily governed by the glycerol fraction of the formulation [26].

Considering the rheological, mechanical, and functional properties, the OPC/Gly-70/30 formulation was selected as the base matrix for subsequent functionalization. This formulation consistently presented the highest viscoelastic moduli in both the frequency-sweep and temperature ramp tests, together with the highest Young’s modulus and tensile strength, indicating a higher stiffness and mechanical resistance compared with the other formulations. Although its elongation at break was lower than that of the 60/40 formulation, as expected for the formulation with the lowest glycerol content, it also showed the lowest soluble matter loss while maintaining a water uptake capacity comparable to the other formulations. Thus, the selection was based on considering water uptake capacity together with soluble matter loss and mechanical performance, rather than on maximizing a single property. The 70/30 formulation provided a suitable compromise between water uptake, resistance to material loss, stiffness, and tensile strength, while retaining sufficient extensibility for subsequent processing. Overall, these results supported the selection of the 70/30 formulation as the most suitable starting matrix for subsequent functionalization, as it combined functional performance with greater mechanical resistance and lower soluble matter loss. This formulation was therefore selected to provide a consistent base matrix for evaluating the effect of injection molding after chemical functionalization with EDTAD.

### 3.2. OPC+EDTAD/Gly Systems Characterization

The effect of EDTAD functionalization was evaluated by comparing the native and functionalized OPC/Gly-70/30 systems (doughs and bioplastics).

#### 3.2.1. Functionalized OPC (OPC+EDTAD): FTIR Analysis

After acylation of OPC, FTIR analysis was first performed to assess the chemical modification of OPC with EDTAD prior to processing (Figure 4). Both the native and functionalized samples exhibited the characteristic absorption bands of protein-based materials, including the broad O–H/N–H stretching band around 3300 cm^−1^, the C–H stretching band near 2930 cm^−1^, and the characteristic Amide I, II, and III bands [27]. The main differences between spectra were observed in the carbonyl region (1750–1600 cm^−1^), where the functionalized sample showed an increase in the absorption intensity around 1650 cm^−1^, which may reflect changes in carbonyl-containing groups following functionalization. This change is consistent with the incorporation of additional carbonyl-containing groups during the acylation reaction between EDTAD and the free amino groups of the protein, potentially resulting in the formation of new amide bonds and pendant carboxyl groups [28,29].

In addition, slight changes were observed in the 1300–1000 cm^−1^ region, assigned mainly to C–O stretching vibrations. Although this region also contains contributions from glycerol and carbohydrate moieties, the increased band intensity after functionalization is consistent with the presence of additional oxygen-containing functional groups introduced by EDTAD. Overall, the spectral changes observed in both regions are consistent with the chemical modification of oat proteins by EDTAD and support the proposed introduction of additional carboxyl-containing groups, in agreement with previous studies on acylated protein-based materials [28,29]. However, FTIR analysis provides qualitative rather than quantitative evidence of the extent of functionalization.

#### 3.2.2. DMA

As shown in Figure 5A, both the functionalized and native OPC/Gly doughs showed a typical viscoelastic response for protein-based systems, with both the storage modulus (E′) and loss modulus (E″) increasing with frequency and E′ remaining higher than E″ throughout the studied range. However, the functionalized formulation displayed consistently lower viscoelastic moduli than the native dough. A similar behavior was observed in the temperature ramp tests (Figure 5B), where the functionalized dough exhibited lower E′ and E″ values over the entire temperature range together with a slight shift of the tan δ peak towards lower temperatures, suggesting an earlier relaxation process. These results demonstrate a reduction in the viscoelastic moduli of the dough following EDTAD functionalization, suggesting lower rigidity prior to thermal processing. This performance may be associated with the incorporation of additional carboxyl-containing groups onto the protein backbone, which increases the hydrophilicity of the protein matrix and could promote greater chain mobility, thereby weakening intermolecular protein interactions in the unprocessed system [29].

Interestingly, the opposite trend was observed after injection molding (Figure 5C,D). The functionalized bioplastics presented higher viscoelastic moduli than the corresponding native materials in both the frequency-sweep and temperature ramp tests, although the differences were less pronounced than those observed for the doughs. These results indicate that the effect of EDTAD functionalization depends on the processing stage. While the additional polar groups soften the protein network before processing, the subsequent injection molding step may modify the viscoelastic response of the functionalized system. Under the combined action of temperature, pressure, and shear, the additional functional groups introduced during EDTAD treatment could promote the establishment of further intermolecular interactions within the protein network. Moreover, because EDTAD is a bifunctional dianhydride capable of reacting with amino groups from different protein chains, it could potentially contribute to intermolecular interactions or bridging, in addition to introducing pendant carboxyl groups, unlike monofunctional acylating agents such as succinic anhydride [28,30]. Accordingly, the higher viscoelastic modulus observed after injection molding may be associated with the development of a more cohesive or interconnected protein structure in the functionalized bioplastics. However, this interpretation remains a proposed mechanism, as the DMA results alone do not directly demonstrate crosslinking or intermolecular bridging. This opposite rheological response before and after injection molding constitutes one of the main findings of this work.

#### 3.2.3. Water Uptake Capacity and Soluble Matter Loss

As shown in Table 3, unexpectedly, the EDTAD-functionalized bioplastic showed a significantly lower water uptake capacity (WUC) than its native counterpart, whereas no significant differences were observed in soluble matter loss (SML). This result contrasts with previous studies reporting enhanced water absorption after protein acylation due to the introduction of additional hydrophilic groups [28,29,30].

The reduced WUC is, however, consistent with the rheological behavior previously observed for the functionalized bioplastics. Although EDTAD introduces additional hydrophilic groups, the higher viscoelastic moduli may reflect changes in the protein structure induced during injection molding. This reinforced structure is expected to restrict matrix swelling and water diffusion, thereby reducing WUC despite the higher intrinsic hydrophilicity of the modified protein. Similar effects have been reported for protein-based materials processed at elevated temperatures, where increased network compactness improves mechanical performance [31]. In the present study, the stronger dependence of WUC on mold temperature observed for the EDTAD-functionalized material may be related to the changes introduced by functionalization, although this mechanism was not directly investigated.

In contrast, the absence of significant differences in SML suggests that soluble matter loss was mainly governed by the glycerol content, which remained constant in both formulations, rather than by protein functionalization itself [26].

Since the functionalized OPC-based bioplastic exhibited a lower WUC than the native OPC-based material when processed at 120 °C, the influence of mold temperature on the absorbent performance of both systems was subsequently investigated. As shown in Figure 6, the functionalized 70/30 EDTAD bioplastic presented a strong dependence of WUC on mold temperature, decreasing progressively from approximately 232% at 60 °C to about 115% at 140 °C. In contrast, the WUC of the native OPC/Gly-70/30 formulation remained nearly constant (approximately 150–160%) over the same temperature range. A two-way ANOVA revealed significant effects of both functionalization and mold temperature on WUC, as well as a significant functionalization × mold temperature interaction (*p* < 0.001). This significant interaction indicates that the effect of mold temperature on WUC depended on the functionalization of the OPC-based bioplastic. As indicated by the different uppercase and lowercase letters in Figure 6, the functionalized and native formulations differed significantly at 60, 80, 120, and 140 °C, whereas no significant difference was observed at 100 °C.

The different WUC trends observed with increasing mold temperature for the native and EDTAD-functionalized formulations suggest that the effect of processing temperature may depend on the functionalization state of the material. Increasing the mold temperature may promote structural rearrangements or a more compact protein matrix in the functionalized bioplastics, potentially restricting water diffusion despite the higher intrinsic hydrophilicity introduced by EDTAD [31]. However, these structural changes were not directly characterized in the present study. Consequently, the beneficial effect of functionalization on WUC was most pronounced at the lowest mold temperature, where the functionalized OPC-based bioplastics showed substantially higher WUC values than both the native OPC-based material and the functionalized samples processed at higher temperatures. These results highlight mold temperature as an important processing parameter affecting the water uptake of the functionalized material. It should be noted that WUC was determined after 24 h of immersion and therefore reflects the water uptake achieved under the specific experimental conditions and period evaluated. While these results demonstrate the enhanced water uptake capacity of the functionalized material, further characterization of water-absorption kinetics, water retention, and dimensional stability after swelling would be required to fully assess its performance for specific absorbent applications.

The soluble matter loss (SML) of the native and EDTAD-functionalized OPC/Gly-70/30 bioplastics is presented in Table 4. The two-way ANOVA revealed significant effects of functionalization (*p* = 0.009) and mold temperature (*p* < 0.001) on SML, as well as a significant functionalization × mold temperature interaction (*p* < 0.001). Post hoc comparisons showed that SML was significantly higher for the EDTAD-functionalized material than for the native material at 60 °C, whereas no significant differences between formulations were detected at higher mold temperatures. Within the EDTAD-functionalized material, SML at 60 °C was significantly higher than at the other mold temperatures, while no significant differences were observed among 80–140 °C. For the native material, significant differences were only detected between 80 and 140 °C. Therefore, considering both the SML and WUC results, 80 °C was selected as the processing temperature for subsequent mechanical characterization. Although processing at 60 °C resulted in the highest WUC, the significantly higher SML observed at this temperature compared with 80 °C indicated a greater loss of soluble material. In contrast, increasing the temperature above 80 °C did not provide an advantage in terms of SML, while WUC progressively decreased. Thus, 80 °C provided a suitable compromise between water uptake and resistance to soluble matter loss and was therefore selected as the processing condition for further characterization, rather than being considered an experimentally optimized temperature.

The selected functionalized bioplastics exhibited a Young’s modulus of 10.88 ± 0.37 MPa, a tensile strength of 0.13 ± 0.04 MPa, and an elongation at break of 1.59 ± 0.42%. These values were significantly lower than those obtained for the corresponding native bioplastics (Table 1), highlighting a clear trade-off between the enhanced water uptake and mechanical performance of the functionalized material. This behavior was accompanied by the higher water uptake capacity of the functionalized systems. This increase in water uptake is consistent with the increased affinity of the matrix for water and the resulting changes in molecular mobility and intermolecular interactions. Similar behavior has been reported for functionalized protein-based bioplastics. In particular, acylation of soy protein with EDTAD was reported to substantially increase water uptake while decreasing Young’s modulus, demonstrating that enhanced water absorption can be accompanied by a reduction in mechanical stiffness [30]. These findings therefore suggest that increasing the water-absorbing capacity of protein-based materials can involve a compromise with their mechanical performance.

From a practical perspective, the mechanical properties obtained in the present study indicate that the functionalized material is not intended to serve as a load-bearing or mechanically demanding bioplastic. Instead, its potential lies in applications where water uptake is a primary functional requirement and only limited mechanical loads are expected. Nevertheless, the low tensile strength (0.13 ± 0.04 MPa) and elongation at break (1.59 ± 0.42%) indicate that the material would have limited resistance to deformation and handling, particularly under wet conditions. Therefore, its practical applicability as an absorbent material would require consideration of the mechanical demands of the specific application. Further optimization of the balance between water uptake and mechanical performance, for example, through adjustment of the functionalization degree, formulation, or processing conditions, would be necessary before application-specific performance can be established.

## 4. Conclusions

To the best of our knowledge, this work is the first to report the production of oat protein-based bioplastics by injection molding and to study how EDTAD functionalization and thermomechanical processing affect their rheological, mechanical, and water-absorption properties. Among the formulations evaluated, OPC/Gly-70/30 was selected as the base formulation based on its combination of properties, exhibiting higher viscoelastic moduli and tensile strength, together with the lowest soluble matter loss and a water uptake capacity comparable to the other formulations. This combination supported its selection as the base matrix for subsequent functionalization.

Functionalization with EDTAD resulted in spectral changes in the FTIR spectrum consistent with the introduction of additional carboxyl-containing groups, providing qualitative evidence of the chemical modification. The most significant finding of this study is that EDTAD functionalization exhibited opposite effects before and after injection molding. Before processing, EDTAD functionalization reduced the viscoelastic properties of the doughs, whereas after injection molding, the functionalized OPC-based bioplastics exhibited higher viscoelastic moduli than the native OPC-based materials. These results demonstrate that the effect of functionalization on the viscoelastic response depends on the processing stage.

Although the functionalized bioplastics processed at 120 °C showed lower water uptake capacity than the native counterparts, reducing the mold temperature markedly increased water uptake, with the significant functionalization × mold temperature interaction confirming that the response to mold temperature depended on the functionalization state. Considering both the water uptake capacity and soluble matter loss, processing at 80 °C was selected as a suitable processing condition for the functionalized system, providing a compromise between water uptake and resistance to soluble matter loss.

Overall, the combination of EDTAD functionalization and low mold temperature enabled substantially higher water uptake of the oat protein-based bioplastics, although this improvement was accompanied by a reduction in tensile properties. This highlights a clear trade-off between absorbency and mechanical performance and indicates that the functionalized material may have limited suitability for applications requiring high mechanical integrity. The present findings highlight the influence of chemical functionalization and thermomechanical processing on the properties of oat protein bioplastics. Further optimization of the balance between water uptake and mechanical performance, together with characterization of absorption kinetics, water retention, and dimensional stability after swelling, would be required before the suitability of these materials for specific absorbent applications can be established.

## Figures and Tables

**Figure 1:**
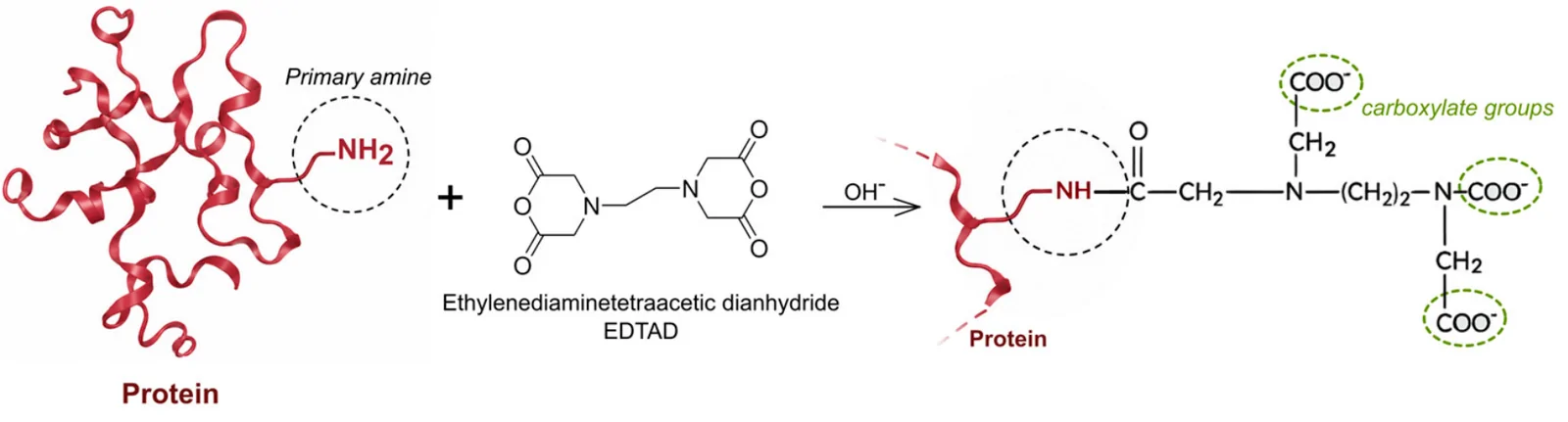
Functionalization of protein by acylation with ethylenediaminetetraacetic dianhydride (EDTAD).

**Figure 2:**
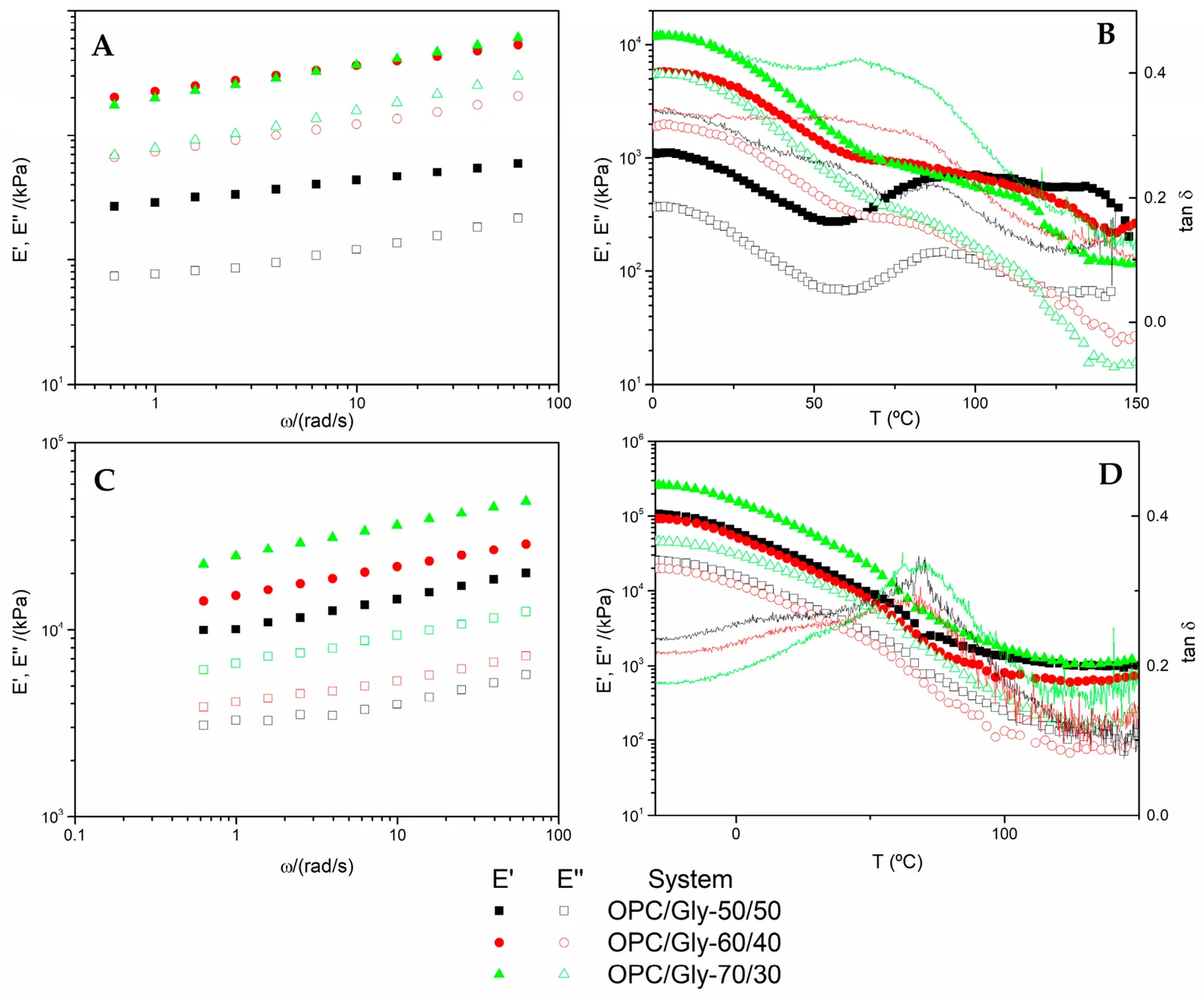
Dynamic mechanical analysis (DMA) of the different OPC/Gly systems: (**A**) frequency-sweep tests of the doughs, (**B**) temperature ramp tests of the doughs, (**C**) frequency-sweep tests of the bioplastics, and (**D**) temperature ramp tests of the bioplastics.

**Figure 3:**
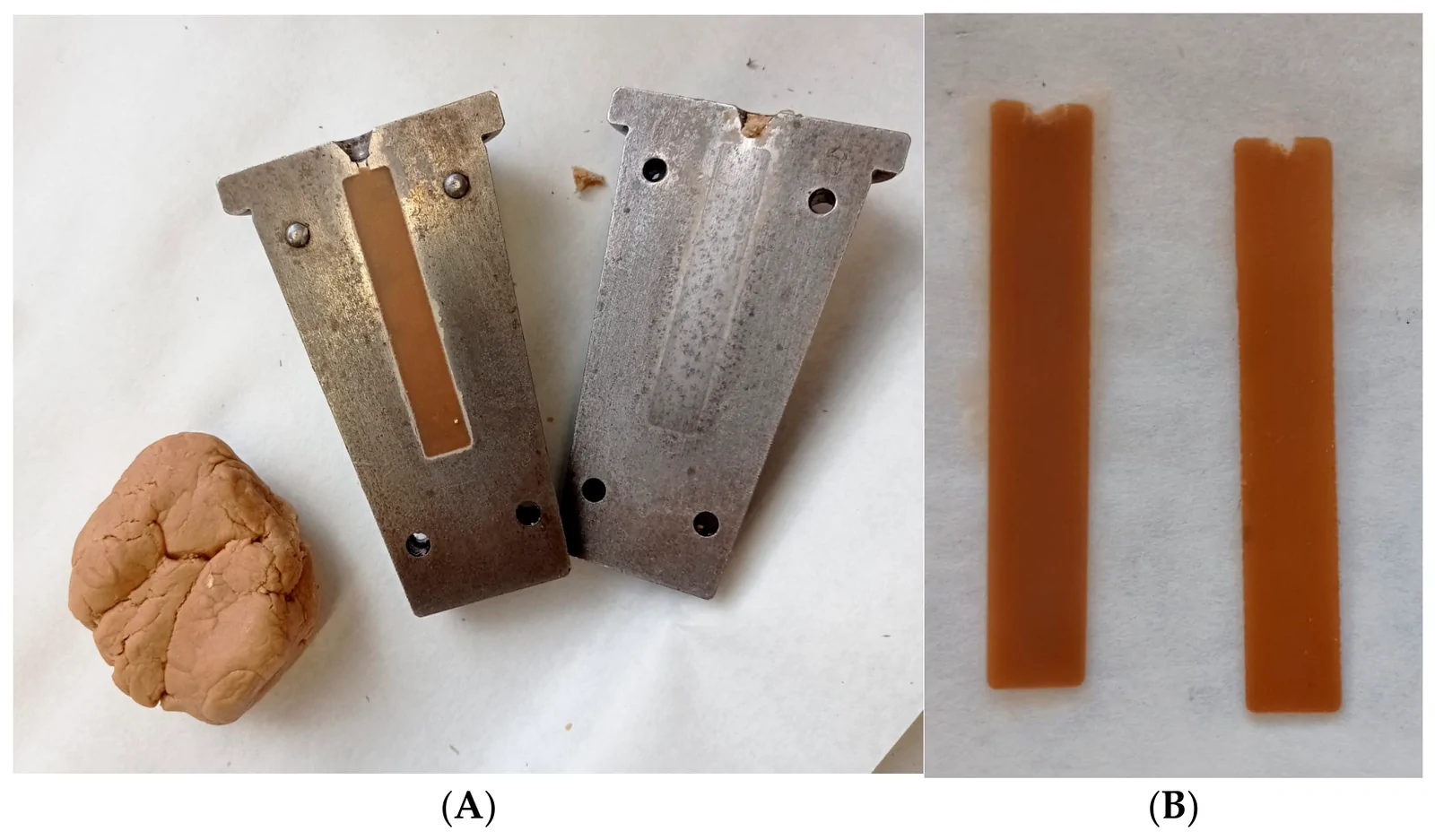
Processing of oat protein concentrate (OPC) into bioplastics by injection molding: (**A**) OPC/Gly dough used as feed material for injection molding and the corresponding mold cavity. (**B**) Injection-molded rectangular specimens used for characterization (60 mm × 10 mm × 1 mm).

**Figure 4:**
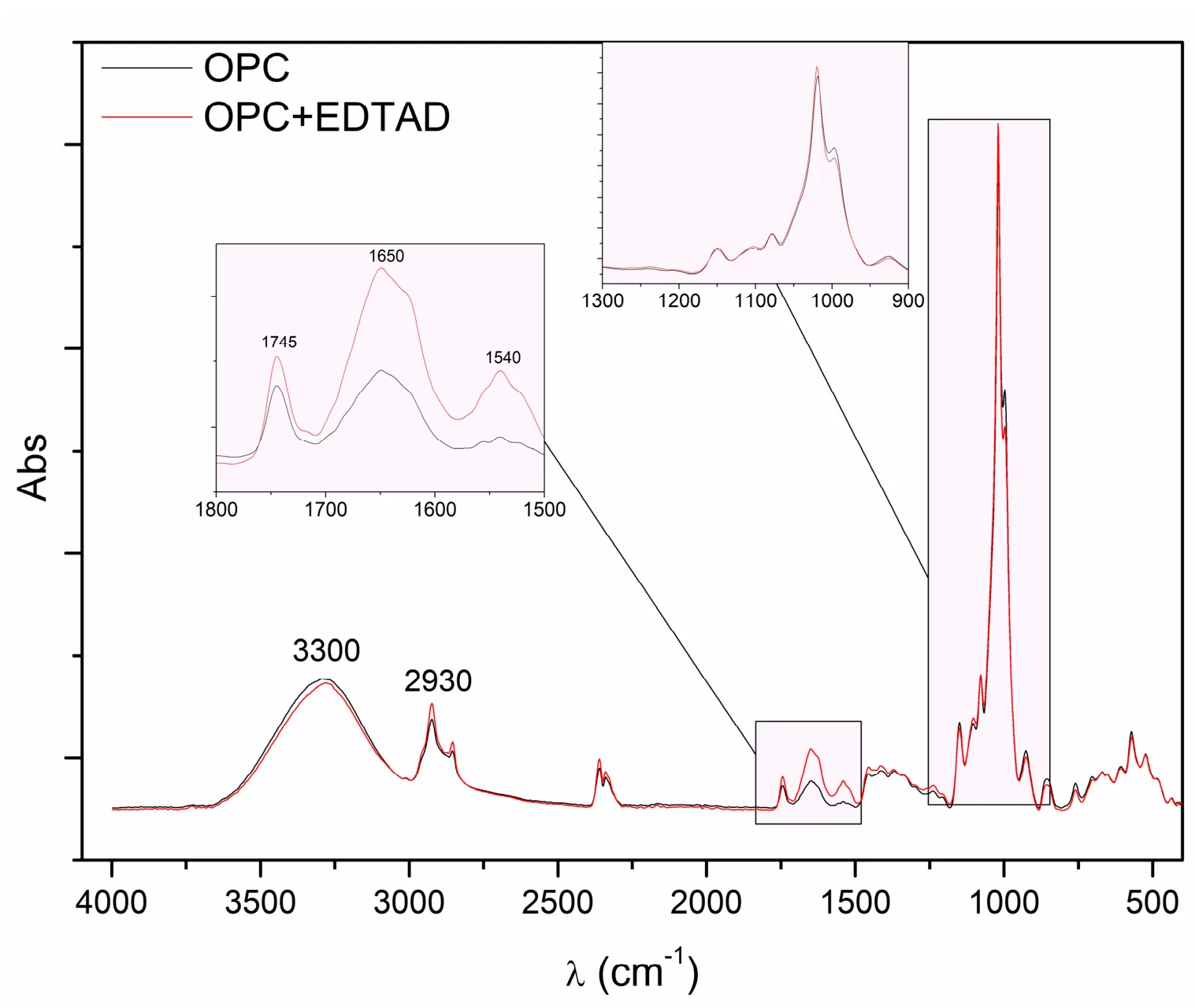
FTIR spectra of native and ethylenediaminetetraacetic dianhydride (EDTAD)-functionalized oat protein concentrate (OPC).

**Figure 5:**
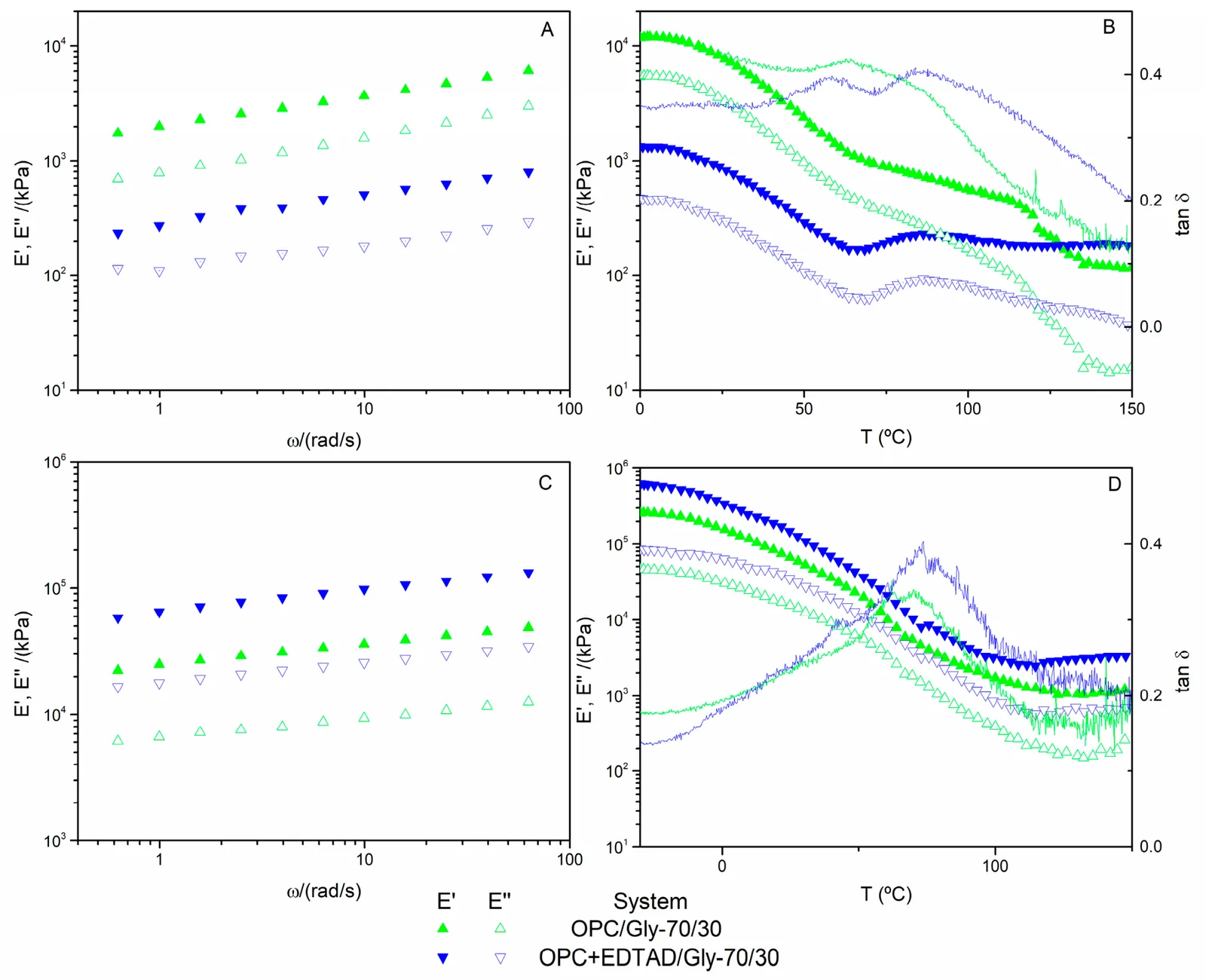
Dynamic mechanical analysis (DMA) of native and ethylenediaminetetraacetic dianhydride (EDTAD)-functionalized OPC/Gly-70/30 systems: (**A**) frequency-sweep tests of the doughs, (**B**) temperature ramp tests of the doughs, (**C**) frequency-sweep tests of the bioplastics, and (**D**) temperature ramp tests of the bioplastics.

**Figure 6:**
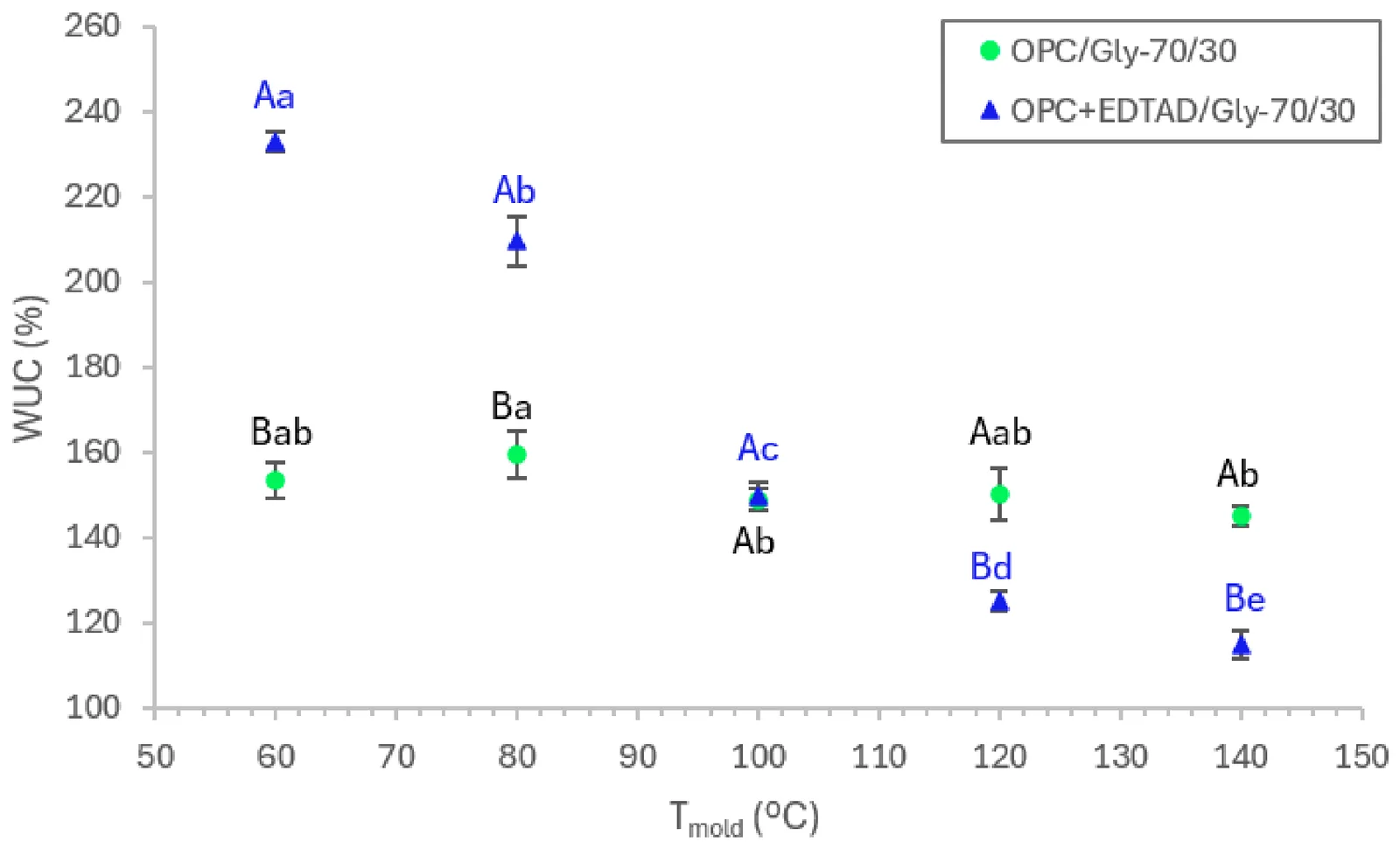
Effect of mold temperature and functionalization with ethylenediaminetetraacetic dianhydride (EDTAD) on the water uptake capacity (WUC) of Oat Protein Concentrate (OPC)/Glycerol (Gly)-70/30 bioplastics. Data are presented as mean ± standard deviation. Different uppercase letters indicate significant differences between formulations at each molding temperature, while different lowercase letters indicate significant differences among molding temperatures within each formulation (*p* < 0.05).

**Table 1:** Tensile properties (Young’s modulus (E), tensile strength (σ_max_), and elongation at break (ε_max_)) of the different Oat Protein Concentrate (OPC)/Glycerol (Gly) bioplastics.

System	E (MPa)	σ_max_ (MPa)	ε_max_ (%)
OPC/Gly-50/50	18.7 ± 1.5 ^b^	0.29 ± 0.02 ^b^	2.00 ± 0.19 ^b^
OPC/Gly-60/40	17.5 ± 1.2 ^b^	0.32 ± 0.03 ^b^	2.69 ± 0.30 ^a^
OPC/Gly-70/30	36.0 ± 2.0 ^a^	0.52 ± 0.04 ^a^	1.92 ± 0.24 ^b^

Values are presented as mean ± standard deviation (*n* = 6). Different letters within the same column indicate significant differences between samples (*p* < 0.05).

**Table 2:** Water uptake capacity (WUC) and soluble matter loss (SML) of the different OPC/Gly bioplastics.

System	WUC (%)	SML (%)
OPC/Gly-50/50	168 ± 6.08 ^ab^	47.3 ± 1.29 ^a^
OPC/Gly-60/40	175 ± 9.60 ^a^	45.7 ± 0.795 ^a^
OPC/Gly-70/30	161 ± 7.78 ^b^	36.3 ± 1.82 ^b^

Values are presented as mean ± standard deviation (*n* = 6). Different letters within the same column indicate significant differences between samples (*p* < 0.05).

**Table 3:** Water uptake capacity (WUC) and soluble matter loss (SML) of native and EDTAD-functionalized Oat Protein Concentrate (OPC)/Glycerol (Gly)-70/30 bioplastics.

System	WUC (%)	SML (%)
OPC/Gly-70/30	160.8 ± 2.7 ^a^	34.63 ± 0.19 ^a^
OPC+EDTAD/Gly-70/30	125.1 ± 2.2 ^b^	33.81 ± 0.75 ^a^

Values are presented as mean ± standard deviation (*n* = 3). Different letters within the same column indicate significant differences between samples (*p* < 0.05).

**Table 4:** Soluble matter loss (SML) of native and ethylenediaminetetraacetic dianhydride (EDTAD)-functionalized Oat Protein Concentrate (OPC)/Glycerol (Gly)-70/30 bioplastics at different mold temperatures (T_mold_).

T_mold_ (°C)	OPC/Gly-70/30	OPC+EDTAD/Gly-70/30
60	35.29 ± 0.65 ^abB^	39.87 ± 1.27 ^aA^
80	35.59 ± 0.82 ^aA^	35.50 ± 0.22 ^bA^
100	35.19 ± 0.52 ^abA^	34.53 ± 1.11 ^bA^
120	34.63 ± 0.19 ^abA^	33.81 ± 0.75 ^bA^
140	33.70 ± 0.32 ^bA^	34.68 ± 0.86 ^bA^

Values are presented as mean ± standard deviation (*n* = 3). Different lowercase letters within the same column indicate significant differences among mold temperatures for the same formulation (*p* < 0.05). Different uppercase letters within the same row indicate significant differences between native and EDTAD-functionalized bioplastics processed at the same mold temperature (*p* < 0.05).

## Data Availability

The original contributions presented in this study are included in the article. Further inquiries can be directed to the corresponding author.

## Supplementary Materials

The following supporting information can be downloaded at https://www.mdpi.com/article/10.3390/polym18192338/s1. Figure S1: Representative tensile stress–strain curves of injection-molded OPC/Gly bioplastics with different protein/glycerol ratios: OPC/Gly-50/50, OPC/Gly-60/40, and OPC/Gly-70/30. One representative curve from the six tested specimens is shown for each formulation.

## Abbreviations

The following abbreviations are used in this manuscript:

Abs	Absorbance
ANOVA	Analysis of variance
DMA	Dynamic mechanical analysis
E	Young’s modulus
E′	Storage modulus
E″	Loss modulus
EDTAD	Ethylenediaminetetraacetic dianhydride
FTIR	Fourier transform infrared spectroscopy
Gly	Glycerol
OP	Oat protein
OPC	Oat protein concentrate
SML	Soluble matter loss
tan δ	Loss tangent
WUC	Water uptake capacity
ε_max_	Elongation at break
λ	Wavelength
σ_max_	Tensile strength
ω	Frequency
